# Ether à go-go potassium channel expression in soft tissue sarcoma patients

**DOI:** 10.1186/1476-4598-5-42

**Published:** 2006-10-05

**Authors:** Fernanda Mello de Queiroz, Guilherme Suarez-Kurtz, Walter Stühmer, Luis A Pardo

**Affiliations:** 1 Divisão de Farmacologia, Coordenação de Pesquisa, Instituto Nacional do Câncer, Rua André Cavalcanti 37/3° andar, Rio de Janeiro, Brazil; 2 Max-Planck-Institut für Experimentelle Medizin Hermann-Rein-Str. 3, 37075 Göttingen, Germany

## Abstract

**Background:**

The expression of the human Eag1 potassium channel (Kv10.1) is normally restricted to the adult brain, but it has been detected in both tumour cell lines and primary tumours. Our purpose was to determine the frequency of expression of Eag1 in soft tissue sarcoma and its potential clinical implications.

**Results:**

We used specific monoclonal antibodies to determine the expression levels of Eag1 in soft tissue sarcomas from 210 patients by immunohistochemistry. Eag1 was expressed in 71% of all tumours, with frequencies ranging from 56% (liposarcoma) to 82% (rhabdomyosarcoma). We detected differences in expression levels depending on the histological type, but no association was seen between expression of this protein and sex, age, grade or tumour size. Four cell lines derived from relevant sarcoma histological types (fibrosarcoma and rhabdomyosarcoma) were tested for Eag1 expression by real-time RT-PCR. We found all four lines to be positive for Eag1. In these cell lines, blockage of Eag1 by RNA interference led to a decrease in proliferation.

**Conclusion:**

Eag1 is aberrantly expressed in over 70% sarcomas. In sarcoma cell lines, inhibition of Eag1 expression and/or function leads to reduced proliferation. The high frequency of expression of Eag1 in primary tumours and the restriction of normal expression of the channel to the brain, suggests the application of this protein for diagnostic or therapeutic purposes.

## Background

Ion channels play important roles in several cellular functions such as excitability, contraction, cell cycle progression and metabolism [1]. The link between ion channels and disease has received widespread attention in the last decade as mutations in several ion channels have been shown to be responsible for certain neurological disorders [2,3]. Whereas many of these mutations affect well-characterised channels of the nervous system, little is know about the effects in non-excitable cells. The voltage-activated potassium channel ether à go-go (Eag1) has recently gained increased interest because of its potential oncogenic role [4,5]. We and others [4] have shown that the Eag1 channel is involved in cell-cycle progression of tumour cells, and that a significant reduction in the cell proliferation of these cell lines could be achieved by inhibiting Eag1 expression using antisense oligonucleotides. It has also been reported that expression of this channel in Chinese hamster ovary cells (CHO) increased both metabolic activity and proliferation rate. In an immunodeficient mouse model, Eag1-transfected CHO cells caused rapidly growing tumours, while wild type CHO cells were much less aggressive, suggesting that Eag1 expression confers a selective advantage to cancer cells [4]. Recently, functional expression of Eag1 was found in samples of cervix carcinoma from patients, while the channel was absent in normal tissue [5].

Another example of the relationship of Eag1 with the cell cycle is the expression of the channel in myoblasts, but not in adult human skeletal muscle. This suggests that Eag1 expression is a cell-cycle related phenomenon, since myoblast fusion is intimately coupled to irreversible cell cycle arrest [6]. Together, these data point to a direct involvement of Eag1 channels in cell proliferation and suggest that these channels participate in the transformation of normal cells into tumour cells. Because of their oncogenic properties, their restricted distribution in normal tissue and ubiquitous expression in tumour cells [4,7], Eag1 potassium channels have gained recent interest as potential targets for cancer detection and therapy.

Soft tissue sarcomas are relatively rare (less than 1% of all cancers) and represent a highly heterogeneous group of tumours. More than 40% of new sarcoma patients die from the disease each year [8]. The relatively small number of cases and great diversity in histopathologic presentation, anatomic site and biological behaviour has made a comprehensive understanding of these disease entities extremely difficult. Clinicians and patients are still faced with limited options of chemotherapy, surgery and radiation therapy, with only modest improvement in survival and cure. While surgery and radiation therapy can be effective in soft tissue sarcomas that manifest a more regional biology, sarcomas with a more systemic biology benefit little from the currently available therapeutic tools. In the present work we studied the expression of Eag1 in soft tissue sarcomas of patients because the normal cells that give rise to these tumours do not express the Eag1 channel [4,6].

## Results

### Efficiency of Eag1 detection depends on the duration of storage

**Figure 1:**
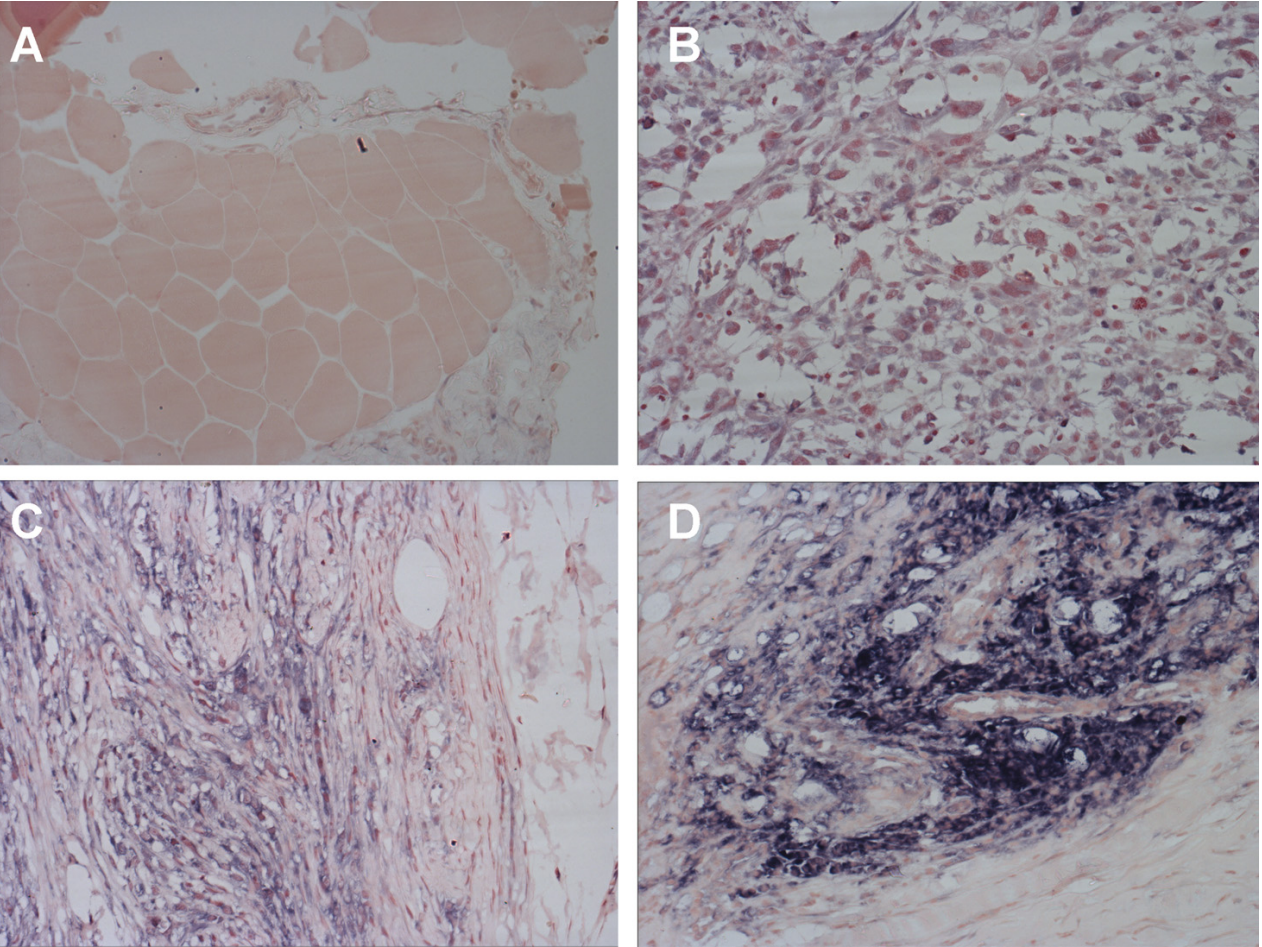
**Typical staining of tumour samples with a single-chain antibody against Eag1**. Eag1 shows homogeneous cytoplasmic staining with perinuclear localization. Shown are representative examples for the different intensities of Eag1 staining leading to the scoring of 0 (A, morphologically non-malignant skeletal muscle from a rhabdomyosarcoma case), 1+ (B, malignant fibrous histiocytoma), 2+ (C, leiomyosarcoma) and 3+ (D, rhabdomyosarcoma) used for further analysis. Magnification: 20×.

Samples from 210 patients with soft tissue sarcoma were stained for Eag1 expression, from which positive signals were observed with high frequency. Eag1 immuno-reactivity was preferentially cytoplasmic with predominant perinuclear localization consistent with results reported by others [9]. Figure 1 shows examples of the different intensities of Eag1 staining leading to the classifications (0, 1+, 2+ and 3+) used for the analysis. The staining intensity for the channel was homogeneous within a preparation and clearly predominant in tumour cells as compared to neighbouring morphologically non-malignant cells (Fig. 1 and Fig. 3).

**Figure 2:**
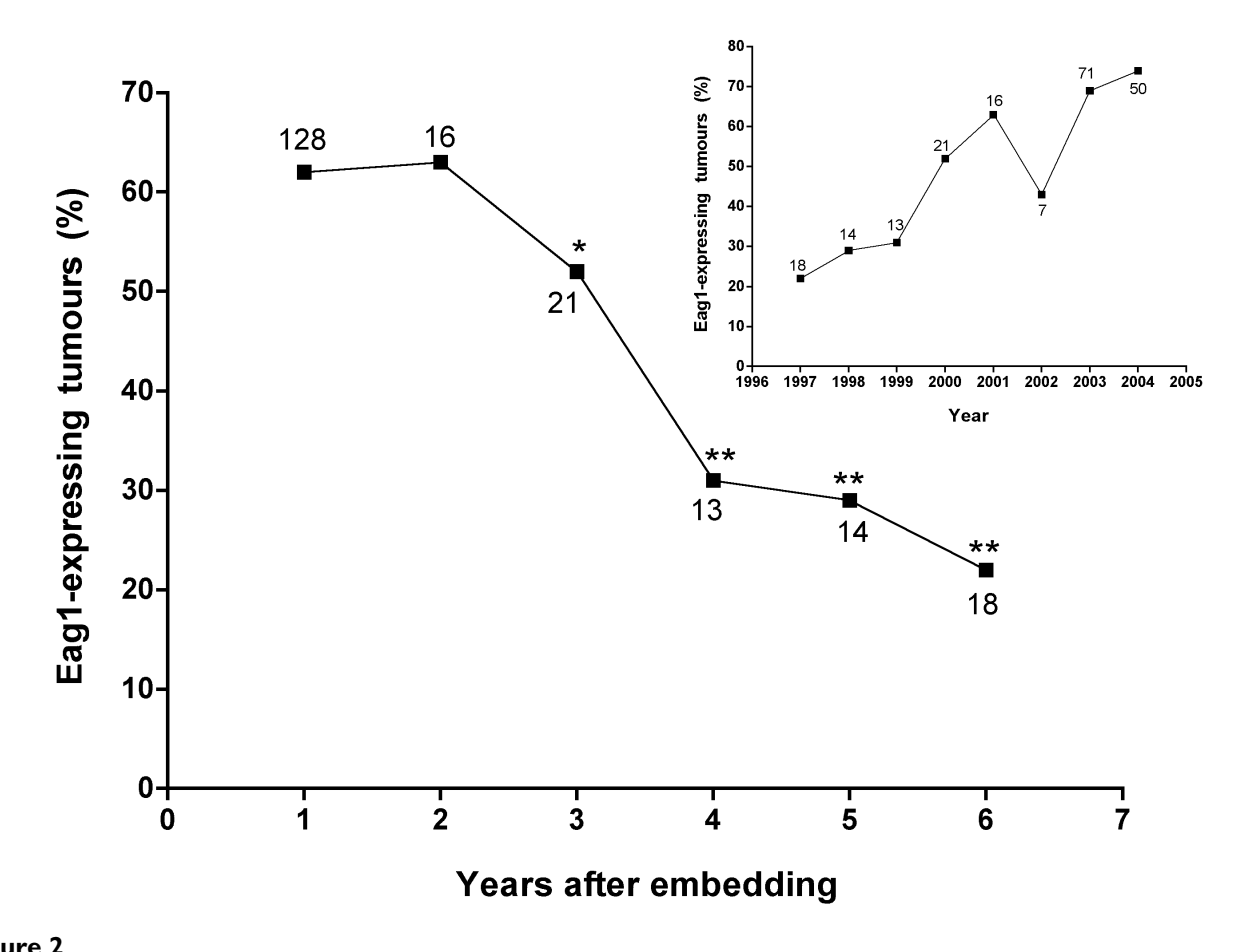
**Preservation of Eag1 antigen in paraffin-embedded tissue**. The percentage of Eag1-expressing tumours decreases with the elapsed storage time of samples before the immunohistochemistry procedure was performed. Insert: The percentage of Eag1-expressing tumours increases over the years (1997–2004) indicating that the Eag1 antigen is not stable in paraffin-embedded tissue for long periods of time. The numbers indicate the number of cases tested for each data point. *p < 0.01, **p < 0.001

**Figure 3:**
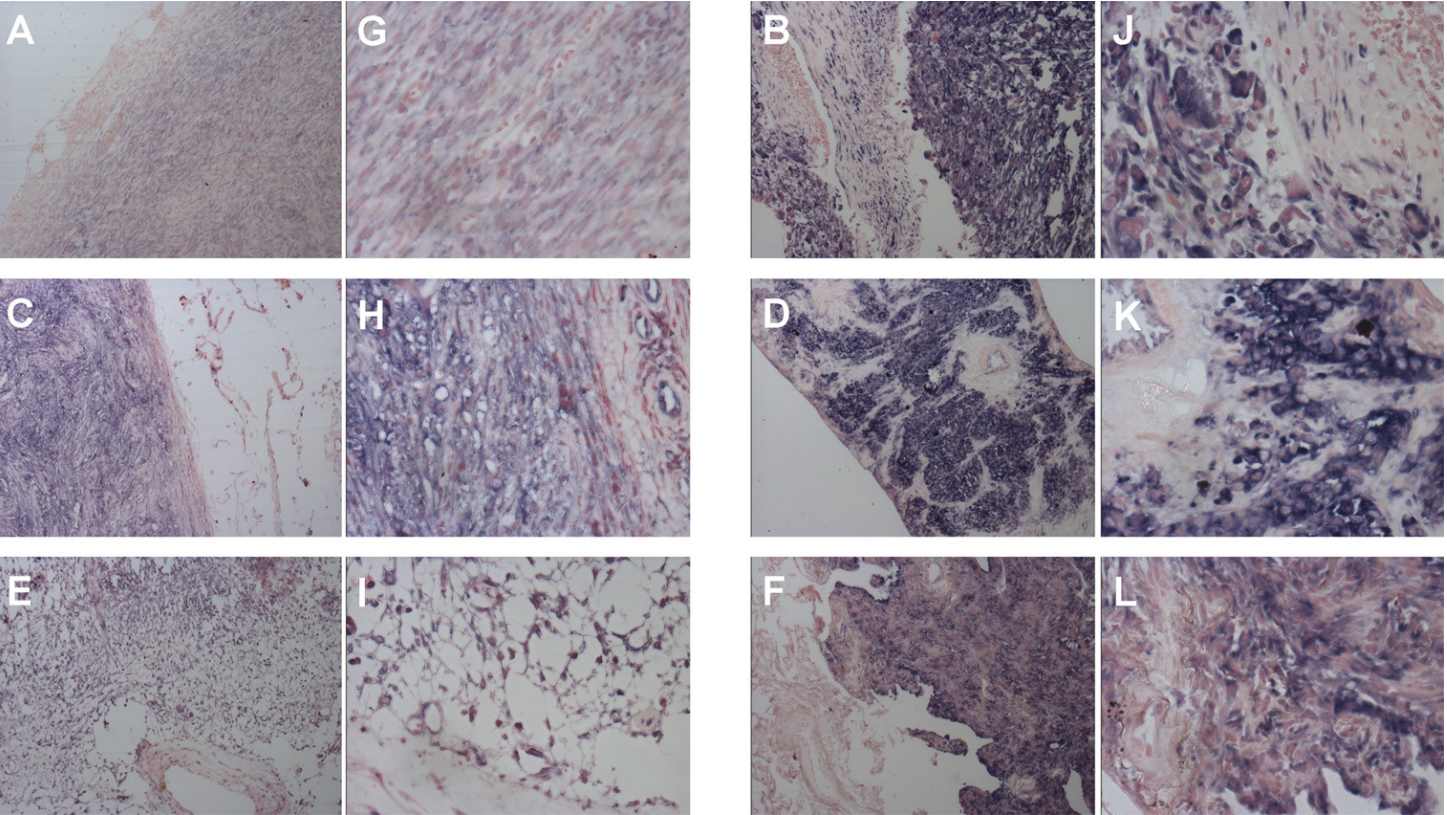
**Representative images of normal and tumour tissue**. Alkaline phosphatase staining using anti-Eag1 antibody. Examples of fibrosarcoma (A, G), leimyosarcoma (B, H), liposarcoma (C, I), malignant fibrous histiocytoma (D, J), rhabdomyosarcoma (E, K) and synovial sarcoma (F, L). In all cases staining of Eag1 was restricted to tumours cells in comparison with the surrounding normal tissue. Magnification: 10× (A-F), 40× (G-L)

When analysing the frequency of positive Eag1 signals, we noticed a correlation between how recently the sample had been obtained and the frequency of positive cases. Figure 2 (*insert*) shows the distribution of Eag1-expressing tumours as a function of the year (between 1997 and 2004) in which the sample had been originally obtained. Most of the cases stemming from 1997 to 1999 were negative, while 50% of cases from 2000 onwards were positive. The limited number of cases from 2002 precludes statistical analysis, but 70% of tumours dating from both 2003 and 2004 were positive. There is an inverse correlation between the time elapsed between storage of the sample and the performance of immunohistochemistry (Fig. 2). Samples archived for up to two years have a homogeneous positive rate for Eag1 and there is no statistically significant difference between samples stored for one or two years. Samples older than three years showed a decrease in positive signals for Eag1, with 22% of positive cases in six-year old samples. This strongly suggests that the Eag1 antigen is labile and becomes undetectable with time. We cannot exclude that the storage conditions could influence antigen stability, and further studies will be required to clarify under which conditions the Eag1 antigen becomes unstable. This particular feature of the Eag1 protein impairs retrospective studies and reduces the effective availability of samples.

### Eag1 is frequently expressed in soft tissue sarcomas

Due to the aforementioned limitations, we used only recently obtained samples for analysis. We stained samples from resective surgical interventions not older than one year (from 2003 and 2004). This limited our collection to 121 samples from 119 sarcoma patients. Table 1 summarises the clinico-pathological features of the cases and the Eag1 channel expression. The frequency of expression of Eag1 in soft tissue sarcoma averaged 71% of the cases studied, with variations between histological types from 56% (liposarcoma) to 82% (rhabdomyosarcoma). No association was detected between Eag1 expression and sex, age, site, grade or tumour size. The expression of Eag1 in the primary site or in metastases (75 and 85%, respectively) was higher than in recidivating tumours (56%), but this difference was not statistically significant. Figure 3 shows representative images of normal (morphologically non-malignant) and neoplastic tissue from all six subtypes of soft tissue sarcoma studied here. We also stained normal tissue from the same patients, which were negative for Eag1 in most of the cases except for restricted populations of normal cells [9].

**Table 1:** Eag1 expression and clinicopathological characteristics.

	**Number of Tumours (%)**	**Eag1 expression (%)**
Sex		
Male	54 (45%)	35 (65%)
Female	67 (55%)	51 (76%)
Age (years)		
< 14	11 (9%)	9 (82%)
15 – 34	19 (16%)	14 (74%)
35 – 64	65 (54%)	46 (71%)
> 65	26 (21%)	17 (65%)
Histology		
Fibrosarcoma	12 (10%)	8 (67%)
Leiomyosarcoma	26 (22%)	19 (73%)
Liposarcoma	32 (26%)	18 (56%)
Malignant fibrous histiocytoma	22 (18%)	18 (81%)
Rhabdomyosarcoma	17 (14%)	14 (82%)
Synovial sarcoma	12 (10%)	9 (75%)
Grade		
Low	70 (58%)	49 (70%)
Moderate	23 (19%)	15 (65%)
High	28 (23%)	21 (75%)
Size		
< 5 cm	74 (61%)	52 (70%)
> 5 cm	47 (39%)	33 (70%)
Site		
Primary	76 (63%)	57 (75%)
Recurrence	32 (26%)	18 (56%)
Metastasis	13 (11%)	11 (85%)

Total	121	86 (71%)

In the attempt to quantify Eag1 staining, we used a simplified adaptation of the HER2 score routinely applied to breast cancers (HercepTest), where the samples are classified as 0, 1+, 2+ and 3+ depending on the number of cells stained and the intensity of staining per cell. We considered staining intensities of 1+ as low Eag1 expression and 2–3+ as high Eag1 expression (Fig. 1). Analysing tumours using this score system, we found high Eag1 expression in 57 patients (47% of the total population), while the other 29 cases showed low Eag1 expression (Table 2). Neither of these two groups showed a correlation between Eag1 expression and sex, grade, site or tumour size, but we observed differences between histological types (Table 2). Rhabdomyosarcoma and synovial sarcoma frequently showed high Eag1 levels (100% and 88% of the tumours (p < 0.01), respectively), while other histological types had homogeneous distribution between low and high Eag1 expression.

**Table 2:** Tumour histology and dichotomised hEAG1 expression.

	**Low Eag1 (%)**	**High Eag1 (%)**
Histology		
Fibrosarcoma	4 (50%)	4 (50%)
Leiomyosarcoma	9 (48%)	10 (52%)
Liposarcoma	5 (28%)	13 (72%)
Malignant fibrous histiocytoma	10 (56%)	8 (44%)
Rhabdomyosarcoma	0 (0%)	14 (100%)
Synovial sarcoma	1 (12%)	8 (88%)

Total	29 (27%)	57 (73%)

### Reduction of Eag1 promotes inhibition of proliferation in soft tissue sarcoma cell lines

To establish a useful model for further studies, we tested Eag1 expression in several well-established sarcoma cell lines (rhabdomyosarcoma – TE-671, A-204 and fibrosarcoma – HT-1080, Hs633t). As positive control we used CHO cells transfected with an Eag1 pTracer vector construct and cells transfected with empty vector as a negative control. These negative control cells did not show Eag1 expression as determined by electrophysiology or immunocytochemistry [4] and contained very low Eag1 mRNA concentration (less than 0.3 copies/1000 cells) in real-time PCR experiments. The positive control (expressing Eag1 under the control of the CMV promoter) showed 39,000 copies per 1000 cells. Such values were of course not reached by any of the tested sarcoma cell lines, but all four lines were clearly positive, with over 140 copies/1000 cells (Table 3).

**Table 3:** Eag1 expression at the RNA level

**Origin**	**Cell line**	**hEag1a**	**hEag2**	**hTFR**
Rhabdomyosarcoma	TE-671	373.1 ± 0.7	0.0004 ± 0.009	2997.5 ± 0.6
Rhabdomyosarcoma	A-204	306 ± 0.6	0.0003 ± 0.01	2923.9 ± 0.7
Fibrosarcoma	HT-1080	141.4 ± 0.5	65.2 ± 0.6	5891.5 ± 0.9
Fibrosarcoma	Hs 633t	147.4 ± 0.9	74.7 ± 0.8	6255.7 ± 0.9
Epithelial	CHO-pt	0.29 ± 0.8	0.0005 ± 0.001	-
Epithelial	CHO-Eag1	39326 ± 0.8	0.0003 ± 0.001	-

**Figure 4:**
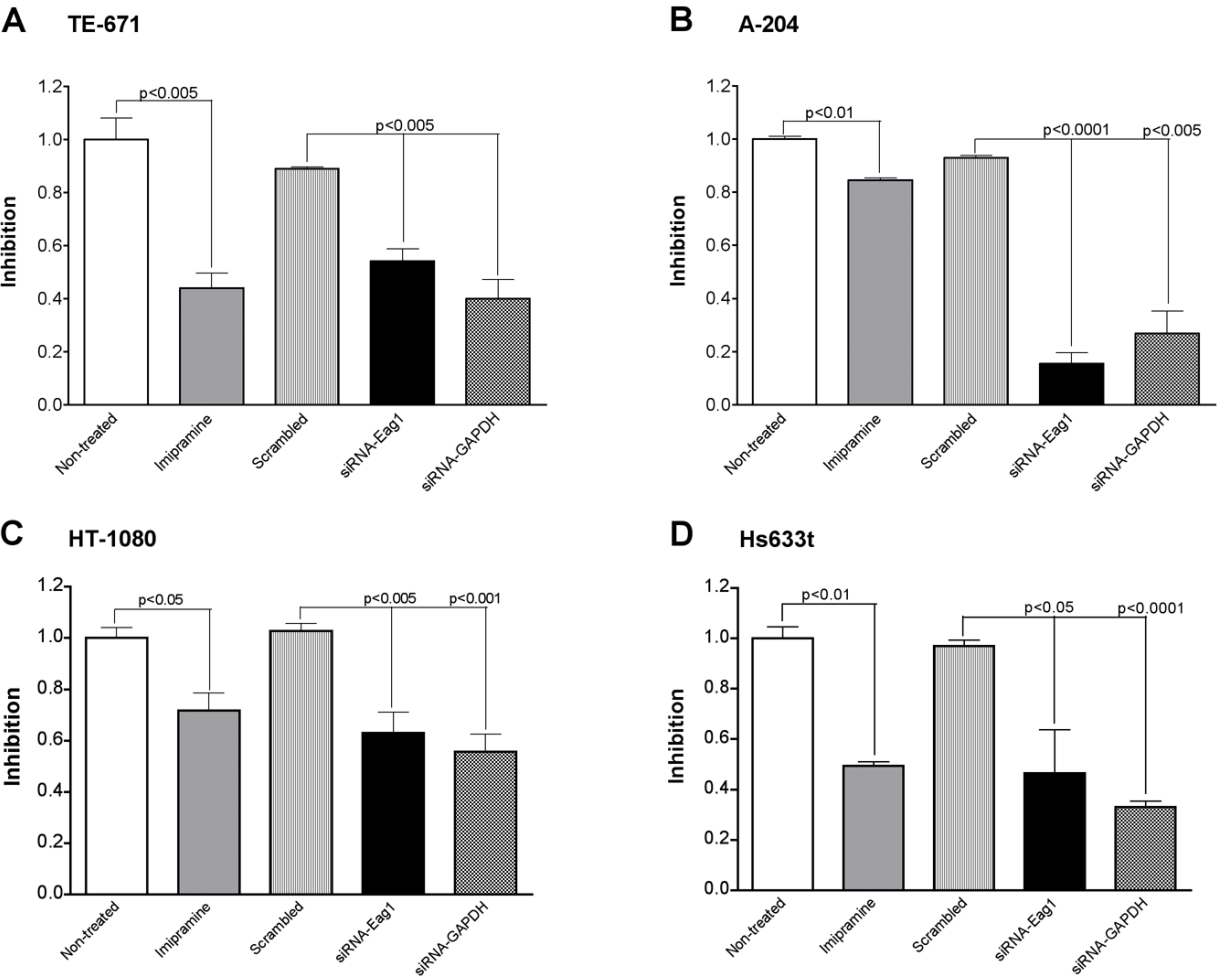
**Inhibition of proliferation of sarcoma cell lines by imipramine or siRNAs**. Soft tissue sarcoma cells (rhabdomyosarcoma – TE-671, A-204 and firbosarcoma – HT-1080, Hs633t) were transfected with siRNAs against Eag1 or were exposed to 10 μM imipramine for 96 hours prior to performing the MTT assay. Data are relative values compared to proliferation in the presence of vehicle and presented as mean ± standard error. Statistical analysis compared imipramine with non-treated cells, and siRNA-Eag1 and -GAPDH against scrambled siRNA molecules, p values are shown.

It has been reported that lowering Eag1 expression in tumour cell lines leads to a reduction of cell proliferation, indicating that Eag1 offers a selective advantage for tumour cells [4]. Therefore, blockage of Eag1 could lead to a reduction of sarcoma cell proliferation, as has been described for melanoma cells [10,11]. Imipramine blocks Eag1 (among other channels) with relative high affinity [12] and we thus studied its effects on the proliferation of the Eag1-positive sarcoma cell lines. We observed a clear reduction of proliferation in all cells incubated in the presence of 10 μM imipramine for 4 days, which is compatible with the hypothesis that Eag1 participates in the proliferation of these cell types (Fig. 4).

Because imipramine is rather non-specific, we used RNA interference as a more specific tool to test if Eag1 participates in the proliferation of soft tissue sarcoma cell lines. We have previously reported the optimization of siRNAs against Eag1 [13]. The reduction of Eag1 mRNA levels by siRNA varies from 70 to 82% depending on the cell line [13]. Figure 4 shows the inhibition of cell proliferation induced by transfection with siRNA in each cell line. As a positive control we used siRNA against GAPDH, which induces a reduction in cell proliferation (or cell metabolism). As a negative control we included a scrambled siRNA. The metabolic activity data were normalized by the values obtained in the presence of the transfection reagent. A significant reduction of proliferation can be observed in all cells treated with siRNA for Eag1 (and for GAPDH). Besides A-204 rhabdomyosarcoma cells, the level of reduction of cell proliferation was the same for both siRNA and imipramine, indicating a possible shared mechanism – namely Eag1 inhibition.

## Discussion

We report here that the voltage-gated potassium channel Eag1 is frequently and robustly expressed in a large proportion of human soft tissue sarcomas. Previous and ongoing studies on this channel have demonstrated that it is expressed in a very selective manner in the brain and a few peripheral cell populations, all of which are epithelial in origin. No expression of the channels has been detected in connective tissues. However, we have found channel expression in 70% of human soft tissue sarcomas and several sarcoma cell lines, but this does not correlate with epidemiological or pathological parameters of the tumours, except for the histological type; rhabdomyosarcoma and synovial sarcoma are strongly positive most frequently.

Unfortunately, samples stored for periods of time longer than two years give unreliable results, probably due to antigen degradation during storage. The liability of the antigen under the archival conditions is not unique for Eag1. An apparent reduction in signal was observed for oestrogen receptor in ten breast cancer samples that were re-evaluated four years after the first analysis (data not shown). These findings imply that archival samples have to be used cautiously when testing potential clinical correlations of a particular antigen. In the case of Eag1, this precludes retrospective studies spanning periods longer than two years. We have nevertheless initiated the prospective analysis of our cases. Despite the limited time period (and number of cases), we have found that the tumours from patients who died over the two-year follow up period were much more likely to express significant levels of Eag1. Of the 19 registered deaths, tumours from 17 expressed Eag1 highly. We could not extract much information from histological samples such as rhabdomyosarcoma or synoviosarcoma because of their generally high Eag1 expression levels (29% and 17% mortality, respectively, all these cases showed high Eag1 expression). Nevertheless, a detailed analysis of the outcome in the liposarcoma population suggests the association of high Eag1 expression with a bad prognosis. Of the 32 liposarcoma cases, 6 (19%) had died during the two-year follow-up period and 5 of these were high Eag1 expressing tumours. These fatalities correspond to 38% of the total high Eag1 expressing liposarcomas, while 93% of the patients with Eag1 negative tumours survived the follow-up period. We also found an indication that the frequency of recurrent disease was higher in the Eag1 positive population- the incidence of recurrence (13%) in Eag1 positive cases was twice as great as that of the Eag1 negatives (6%). Taken together, our data may indicate an association between Eag1 expression and bad prognosis. Further studies with a longer outcome (5 years) and a higher number of patients will clarify the potential usefulness of Eag1 as a prognostic marker.

Due to the behaviour of the antigen with time, prospective studies will be required to investigate whether there is any correlation between levels of Eag1 expression and clinical outcome and/or response to therapy. Such a correlation would obviously be of great interest, since the channel could then serve as a prognostic marker. The lack of correlation of the channel with epidemiological factors (sex, age, grade and site of tumour) makes it difficult to assess a potential prognostic value of Eag1 expression at this stage. However, it should be kept in mind that the histopathological samples showing the highest abundance of Eag1 expression, rhabdomyosarcoma and synovial sarcoma (p < 0.01), are particularly aggressive tumours. There was also a clear difference between tumour and morphologically non-malignant tissue in the same slides with respect to Eag1 expression, the non-malignant tissue always being negative. This could open the possibility of the use of Eag1 as a tool for the differential diagnosis of malignant transformation together with morphological criteria.

It is still remains possible that the frequency of negative tumours (29%) in our series is due to poor detection sensitivity of the antibody and that the actual incidence of Eag1 expression is even higher. Our Eag1 detection procedure relied on a directly coupled single chain antibody. This reduces the probability of false positives that can arise with the use of secondary antibodies and developing systems, but simultaneously looses two multiplicative amplification steps – the secondary antibody and the widely used peroxides polymers. Therefore, while our method is more specific, it is about 60 times less sensitive than other conventional immunohistochemical procedures. The application of methods specifically designed to detect minute quantities of biological macromolecules such as in-situ immuno-PCR [14] will give a clearer estimate of the actual incidence of Eag1 over-expression in soft tissue sarcomas. Another factor increasing the difficulty of analysing Eag1 expression, and more specifically its potential usefulness as a tumour marker, is that Eag1 immunoreactivity is mainly intracellular (Figure 1). Although electrophysiological experiments detected Eag1 currents in tumour cells [5,7], unequivocally indicating surface expression of functional channel proteins, the vast majority of the protein in a particular cell is intracellular. Even the artificially expressing cell line used in this study shows mainly cytoplasmic Eag1 staining, while exhibiting robust Eag1 currents in electrophysiological experiments. We therefore cannot exclude a correlation between plasma membrane Eag1 and clinical outcome.

Among the positive specimens, tumour cells and the surrounding morphologically non-malignant cells can be clearly demarcated. Should Eag1 consistently be a distinct characteristic of tumour cells, this finding presents a potential therapeutic opportunity to distinguish between normal and tumour cells. If at least a fraction of the protein is accessible from the extracellular side, Eag1 may be a good candidate for immunotherapeutical approaches. In vitro experiments in this study showed that several sarcoma cell lines express abundant Eag1 protein and that blockage of the channel by imipramine correlates with a reduction of cell proliferation. Unfortunately, the tricyclic anti-depressant imipramine blocks not only Eag1 but also many different channels such as cardiac and neuronal sodium, calcium and potassium channels [12]. To overcome this problem we used RNA interference, which represents a suitable alternative to functional blockers of channel activity [13,15]. siRNAs for Eag1 have been thoroughly characterized and do not induce non-specific responses [13]. Eag1 siRNA treatment resulted in a reduction of proliferation in all soft tissue sarcoma cell lines (Fig. 4) to an extent comparable to that induced by imipramine.

## Conclusion

Although absent from healthy tissues, Eag1 expression can be detected in 70% soft tissue sarcomas. The frequency of Eag1 expression depends on the histological type, but we found no association with other epidemiological parameters. We could establish a preliminary association of high Eag1 expression and bad prognosis. Additionally, we could show that inhibition of Eag1 expression and/or function reduces the proliferation of several sarcoma cell lines. These findings highlight the role of Eag1 in the proliferation of tumour cells and prompt further studies to validate Eag1 as a potential diagnostic tool and/or anti-cancer ion channel target.

## Methods

### Patients

Two hundred and ten patients from the Brazilian National Cancer Institute (INCA), who had surgery between 1997 and 2004 for resection of soft tissue sarcomas were included in this study. The study was approved by the Ethics Committee at INCA The clinical stage of the surgical specimens was classified according to the three-grade system [16], and the sarcomas were categorised on the basis of histology and immunohistochemistry.

### Anti-Eag1 antibody

A monoclonal antibody anti-Eag1, a fusion protein comprising the single chain antibody fragment derived against the Eag1 potassium channel and bacterial alkaline phosphatase [9] was employed. The antibody was used at a dilution of 1:100 in Tris buffered saline (TBS) with 0.2% bovine serum albumin (BSA, Sigma Chemical Co., St Louis, MO, USA) and 0.25% sodium azide.

### Immunohistochemistry

Formalin-fixed, paraffin-embedded tissue was cut into 4 μm sections and mounted on poly-L-lysine-coated slides. Sections were deparaffinated in xylene and rehydrated in descending alcohols. Antigen retrieval was performed by heating the slides for 30 minutes in a steamer in 10 mM citrate buffer solution pH 6.0. Afterwards, the slides were cooled down to room temperature for at least 30 minutes. Non-specific binding sites were blocked using 10% BSA in TBS for 30 minutes at room temperature. Subsequently, the slides were incubated for 2 hours at 37°C with the antibody. Sections were rinsed with a detection buffer solution containing in mM: 100 Tris-base, 100 NaCl and 5 MgCl2 at pH 8.8. Finally, sections were developed with 5-bromo-4-chloro-3-indolyl phosphate/nitroblue tetrazolium (BCIP/NBT, Roche, Mannheim, Germany) for 20 minutes, slightly counterstained with nuclear fast red (Dako, Glostrup, Denmark), dehydrated and mounted with xylene-based balsam. Slides dating from 1997 until 2002 were stained in July – November 2003, whereas slides prepared in 2003 and 2004 were stained in November – December of the respective year.

### Immunohistochemical evaluation

To achieve a semi-quantitative estimation of expression levels, we used an immunohistochemical score based on the HercepTest for Her2/Neu [17]. Scores were 0 (less than 10% of the tumour cells show staining), 1+ (faint staining in more than 10% of the cells), 2+ (moderate staining in more than 10% tumour cells) and 3+ (strong staining in more than 10% of the cells). The immunohistochemical score was evaluated as negative (0), positive (1) and strongly positive (2 and 3). Examples for each score are shown in Figure 1.

Each stained slide was scored by two independent observers. There were no major disagreements regarding scoring and the average scoring is reported.

### Cell culture

Sarcoma-derived cell lines TE-671 (DSMZ ACC 263), HT-1080 (DSMZ ACC315), and A-204 (DSMZ ACC 250) were obtained from the DSMZ (Braunschweig, Germany). Hs 633T (ECACC 89050201) was obtained from the European Collection of Cell Culture (Salisbury, United Kingdom). All cells were maintained in 125 ml flasks in a humidified atmosphere at 37°C with 5% CO_2 _and passaged every 4–5 days. TE-671, HT-1080 and Hs 633t cells were cultured in Dulbecco's modified Eagle's medium (DMEM, Invitrogen, Karlsruhe, Germany) and A-204 cells were cultured in McCoy's 5A (Invitrogen) containing 10% fetal calf serum (FCS).

### siRNA transfections

Four different siRNAs were designed to target both Eag1 variants with accession numbers NM_172362 and NM_172376, respectively, using the HiPerformance siRNA Design Algorithm. siRNAs (25 nM) were transfected using either Oligofectamine, Lipofectamine (Qiagen) or Dharmacon Transfection Reagent 2 (Dharmacon) transfection reagents in OptiMEM medium (Invitrogen). Cells were plated one day before transfection. The following siRNAs were used: SiRNAs directed against hEag1 (KCNH1, Kv10.1) with the target sequence NM_172362: Kv10.1 nt 1509–1529; Kv10.1-1 nt 236–256; Kv10.1-2 nt 863–883; Kv10.1-3 nt 1793–1813; Kv10.1-4 nt 1022–1042. All siRNAs were synthesized by Qiagen and annealed prior to use, except the commercial Negative Control #1 and the human GAPDH siRNA (Ambion), which we used as negative and positive controls, respectively. The cells were incubated with the siRNA and the transfection reagent for between 6 h and 8 h. Cells were harvested for the experiments 96 hours after the start of transfection. Additionally cells treated only with OptiMEM and Oligofectamine, Lipofectamine or Dharmacon Transfection Reagent 2 were included as controls.

### Real-Time RT PCR

Total RNA was purified from cultures using RNeasy mini kit (Qiagen, Hilden, Germany) First strand cDNA was produced using SuperScript (Invitrogen, Karlsruhe, Germany) with gene-specific and oligo dT as primers. Real-time PCR was performed on the template using the TaqMan system in an AbiPrism 7700 Sequence Detector (Applied Biosystems, Foster City, CA). The oligonucleotides used have been described elsewhere [9]. The human transferrin receptor was used as a control for RNA integrity. The number of PCR cycles to reach detection threshold was used to determine specific mRNA content with a standard curve for interpolation.

### Proliferation studies

Cell proliferation was determined by measuring the metabolic activity via reduction of the tetrazolium salt MTT (3- [4,5-dimethylthiazol-2]-2,5-diphenyltetrazolium bromide; Sigma, Munich, Germany). Cells were plated at a density of 10^4 ^cells/ml and MTT (5 mg/ml) was added to the cultures after 96 hours. Four hours later, the product was solubilised and the reaction stopped using 10% SDS in 0.01 M HCl. After overnight incubation, the colour was read at 562 nm [18]. In some experiments, the actual number of cells was measured in flow cytometry, giving good correlation with the MTT method.

### Statistical analysis

Statistical analysis was performed using the GraphPad Prism^® ^v.4.0 software Differences in sex, age, grade, size, site and tumour histology were assessed using the Chi-square test. We used one-way ANOVA test followed by Bonferroni post test to compare differences that may have arisen in the preservation of samples over the years. Additionally, the unpaired t test was used for the statistical analysis of proliferation data.

## Competing interests

LAP and WS are shareholders of a company interested in the development of ion channel anticancer targets.

## Authors' contributions

FMQ collected the data; all authors participated in the study conception, design and analysis of data, as well as in writing the manuscript.

## References

[B1] Hille B (2001). Ion channels of excitable membranes, Third Edition.

[B2] Bauer CK, Schwarz JR (2001). Physiology of EAG K^+^ channels. J Membr Biol.

[B3] Hatta S, Sakamoto J, Horio Y (2002). Ion channels and diseases. Med Electron Microsc.

[B4] Pardo LA, del Camino D, Sánchez A, Alves F, Brüggemann A, Beckh S, Stühmer W (1999). Oncogenic potential of EAG K^+^ channels. EMBO J.

[B5] Farias LMB, Bermúdez Ocaña D, Díaz L, Larrea F, Avila-Chávez E, Cadena A, Hinojosa LM, Lara G, Villanueva LA, Vargas C, Hernández-Gallegos E, Camacho-Arroyo I, Dueñas-González A, Pérez-Cárdenas E, Pardo LA, Morales A, Taja-Chayeb L, Escamilla J, Sánchez-Peña C, Camacho J (2004). Ether à go-go Potassium Channels as Human Cervical Cancer Markers. Cancer Res.

[B6] Occhiodoro T, Bernheim L, Liu JH, Bijlenga P, Sinnreich M, Bader CR, Fischer-Lougheed J (1998). Cloning of a human ether-a-go-go potassium channel expressed in myoblasts at the onset of fusion. FEBS Lett.

[B7] Meyer R, Schonherr R, Gavrilova-Ruch O, Wohlrab W, Heinemann SH (1999). Identification of ether a go-go and calcium-activated potassium channels in human melanoma cells. J Membr Biol.

[B8] Jemal A, Murray T, Ward E, Samuels A, Tiwari RC, Ghafoor A, Feuer EJ, Thun MJ (2005). Cancer statistics, 2005. CA Cancer J Clin.

[B9] Hemmerlein B, Weseloh RM, Queiroz FM, Knötgen H, Sánchez A, Rubio ME, Martin S, Schliephacke T, Jenke M, Stühmer W, Pardo LA, Heinz-Joachim-Radzun (2006). Overexpression of Eag1 potassium channels in clinical tumour specimens. Mol Cancer.

[B10] Ouadid-Ahidouch H, Le Bourhis X, Roudbaraki M, Toillon RA, Delcourt P, Prevarskaya N (2001). Changes in the K+ current-density of MCF-7 cells during progression through the cell cycle: Possible involvement of a h-ether.a-gogo K^+^ channel. Receptor Channel.

[B11] Gavrilova-Ruch O, Schonherr K, Gessner G, Schonherr R, Klapperstuck T, Wohlrab W, Heinemann SH (2002). Effects of imipramine on ion channels and proliferation of IGR1 melanoma cells. J Membr Biol.

[B12] Garcia-Ferreiro RE, Kerschensteiner D, Major F, Monje F, Stuhmer W, Pardo LA (2004). Mechanism of block of hEag1 K^+^ channels by imipramine and astemizole. J Gen Physiol.

[B13] Weber C, Mello de Queiroz F, Downie B, Sukow A, Stühmer W, Pardo LA (2006). Silencing the activity and proliferative properties of the human Eag1 potassium channel by RNAi. J Biol Chem.

[B14] Cao Y, Kopplow K, Liu GY (2000). In-situ immuno-PCR to detect antigens. Lancet.

[B15] Gurney AM, Hunter E (2005). The use of small interfering RNA to elucidate the activity and function of ion channel genes in an intact tissue. J Pharmacol Toxicol Methods.

[B16] Costa J, Wesley RA, Glatstein E, Rosenberg SA (1984). The grading of soft tissue sarcomas. Results of a clinicohistopathologic correlation in a series of 163 cases. Cancer.

[B17] Cytomation DAKO (2004). Immunohistochemistry and Breast Cancer
Diagnosis, Therapy and Prognosis.

[B18] Mosmann T (1983). Rapid colorimetric assay for cellular growth and survival: application to proliferation and cytotoxicity assays. J Immunol Meth.

